# MicroRNA-222-3p/GNAI2/AKT axis inhibits epithelial ovarian cancer cell growth and associates with good overall survival

**DOI:** 10.18632/oncotarget.13017

**Published:** 2016-11-02

**Authors:** Xiaodan Fu, Yimin Li, Ayesha Alvero, Juanni Li, Qihui Wu, Qing Xiao, Yulong Peng, Yongbin Hu, Xiang Li, Wenguang Yan, Ke Guo, Wenjuan Zhou, Yong Wang, Junwen Liu, Yu Zhang, Gil Mor, Jifang Wen, Gang Yin

**Affiliations:** ^1^ Department of Pathology, Xiangya Hospital, Central South University, Changsha, Hunan Province, China; ^2^ Department of Pathology, School of Basic Medical Sciences, Central South University, Changsha, Hunan Province, China; ^3^ Department of Obstetrics, Gynecology and Reproductive Sciences, Reproductive Immunology Unit, Yale University School of Medicine, New Haven, CT, USA; ^4^ Department of Gynecology, Xiangya Hospital, Central South University, Changsha, Hunan Province, China; ^5^ Department of Rehabilitation, The Third Xiangya Hospital, Central South University, Changsha, Hunan Province, China; ^6^ Department of Internal Neurology, The Third Xiangya Hospital, Central South University, Changsha, Hunan Province, China; ^7^ School of Nursing, Central South University, Changsha, Hunan Province, China; ^8^ Department of Immunology, School of Basic Medical Sciences, Central South University, Changsha, Hunan Province, China; ^9^ Department of Histology and Embryology, School of Basic Medical Sciences, Central South University, Changsha, Hunan Province, China

**Keywords:** ovarian cancer, miR-222-3p, GNAI2, pAKT, cell growth

## Abstract

Ovarian carcinoma is the most lethal gynecologic tumor worldwide. Despite having developed molecular diagnostic tools and targeted therapies over the past few decades, patient survival is still quite poor. Numerous studies suggest that microRNAs are key regulators of many fundamental biological processes, including neoplasia and tumor progression. miR-222 is one of those miRNAs that has attracted much attention for its multiple roles in human diseases, especially cancer. The potential role of microRNAs in ovarian cancer has attracted much attention in recent years. Some of these microRNAs have been suggested as potential therapeutic targets for EOC patients. In this study, we sought to investigate the biologic functions of miR-222-3p in EOC carcinogenesis. Herein, we examined the expression of miR-222-3p in EOC patients, mouse models and cell lines, and found that higher expression of miR-222-3p was associated with better overall survival in EOC patients, and its level was negatively correlated with tumor growth *in vivo*. Furthermore, *in-vitro* experiments indicated that miR-222-3p inhibited EOC cell proliferation and migration, and decreased the phosphorylation of AKT. We identified GNAI2 as a target of miR-222-3p. We also found that GNAI2 promoted EOC cell proliferation, and is an activator of the PI3K/AKT pathway. We describe the characterization of a novel regulatory axis in ovarian cancer cells, miR-222-3p/GNAI2/AKT and its potential application as a therapeutic target for EOC patients.

## INTRODUCTION

Epithelial ovarian carcinoma (EOC) is the most common type of Ovarian cancer (OC), accounting for 90% of the total [1], which is the leading cause of gynecological cancer deaths with a relatively low 5-year survival rate. Although 80–90% of EOC patients initially respond to first-line chemotherapy agents, platinum and paclitaxel, less than 10–15% remain in complete remission and most patients recur within 5 years [2]. Although a number of patients present with high initial responsiveness to chemotherapy, survival rates still remain low due to later drug resistance and cancer recurrence; and by then, fewer therapeutic options are available and the attention focuses on prolonging patient life expectancy [3, 4]. Therefore, it is imperative to elucidate more targeted treatments.

MicroRNAs (miRNAs) consititute a continuously developing class of small (17–25 nucleotides in size) single-stranded non-coding RNA molecules that are highly evolutionarily conserved in a variety of eukaryotic organisms [5]. They can negatively regulate target gene expression primarily by base pairing in a sequence-specific way at the posttranscriptional level, binding to the 3′-untranslated region (3′-UTR) of target gene messenger RNAs (mRNAs), and by inhibiting the protein translation process. In some cases, they directly degrade the target mRNAs.

About 1,100 different miRNAs have been identified in humans, according to microRNA.org updated on 1st Nov 2010. Over the past decade, a growing number of research studies on miRNAs have been carried out, and these show that miRNAs play crucial roles in the regulation of diverse physiologic and pathologic processes, such as cellular proliferation, differentiation and apoptosis [5–7], moreover, dysregulated expression of miRNAs exists not only in many pathologic conditions or diseases, but also in various human cancer types, indicating that aberrant regulation of miRNAs constitutes one of the most important hallmarks of cancer [8, 9]. Whether they function as onco-miRs or tumor suppressor-miRs mainly depends upon cellular context and the target genes, the latter modulating many complicated signaling networks involved in tumor initiation and progression [9, 10].

Among such a large number of miRNAs already identified as being involved in tumorigenesis, miR-221/222 have emerged as key miRNAs, which are encoded in tandem from a single transcript located on human chromosome Xp11.3, with the same seed sequence, and show high sequence identity [11, 12]. miR-222 can play a role as an onco-miR in some cancer types, such as cervical cancer [13], prostate carcinoma [14], since it can target tumor suppressors, such as PTEN [13, 15], p27Kip1 [14], and TIMP3 [15], promoting the proliferation, migration and invasion of cancer cells. Interestingly, it also can function as a tumor suppressor-miR in other cancer types, for example, in erythroblastic leukemia [16], and gastrointestinal stromal tumors [17, 18], miR-222 inhibits cell growth and induces apoptosis by targeting KIT [16, 17] and ETV1 [17]. However, the role of miR-222 in ovarian cancer is not yet clear.

GNAI2 (G protein alpha inhibiting activity polypeptide 2, Galphai2, Giα2) exerts multiple effects in regulating cellular functions, and is correlated with many forms of tumors. In ovarian cancer cells, LPAR1/3-GNAI2 mediates the LPA-stimulated activation of p130Cas, and this LPAR1/3-GNAI2-p130Cas signaling network is intimately associated with invasive migration of ovarian cancer cells [19]. In prostate cancer, siRNAs of GNAI2 slowed OXT-induced migration of PC3 cells [20]; and in tongue squamous cell carcinoma, miR-138 reduced cellular proliferation, arrested cell cycle, and increased apoptosis, at least in part, by targeting GNAI2 [21]. Interestingly, in hepatocellular carcinoma, miR-30d significantly promoted HCC cell invasion and metastasis by targeting GNAI2 [22]. The mechanisms associated with the regulation of GNAI2 expression in cancer cells and specifically in ovarian cancer is poorly understood.

In the present study, we sought to investigate the biologic functions and underlying functional role of miR-222-3p in EOC carcinogenesis. Our data demonstrates that miR-222-3p is a major regulator of GNAI2 expression and its function through its effect on the AKT pathway, a central regulator of cell proliferation and cell death. Therefore, we might identify a novel regulatory axis in EOC cell lines, miR-222-3p/GNAI2/AKT and its potential application as a therapeutic target for EOC patients.

## RESULTS

### Elevated expression of miR-222-3p is associated with improved overall survival of EOC patients

**Figure 1:**
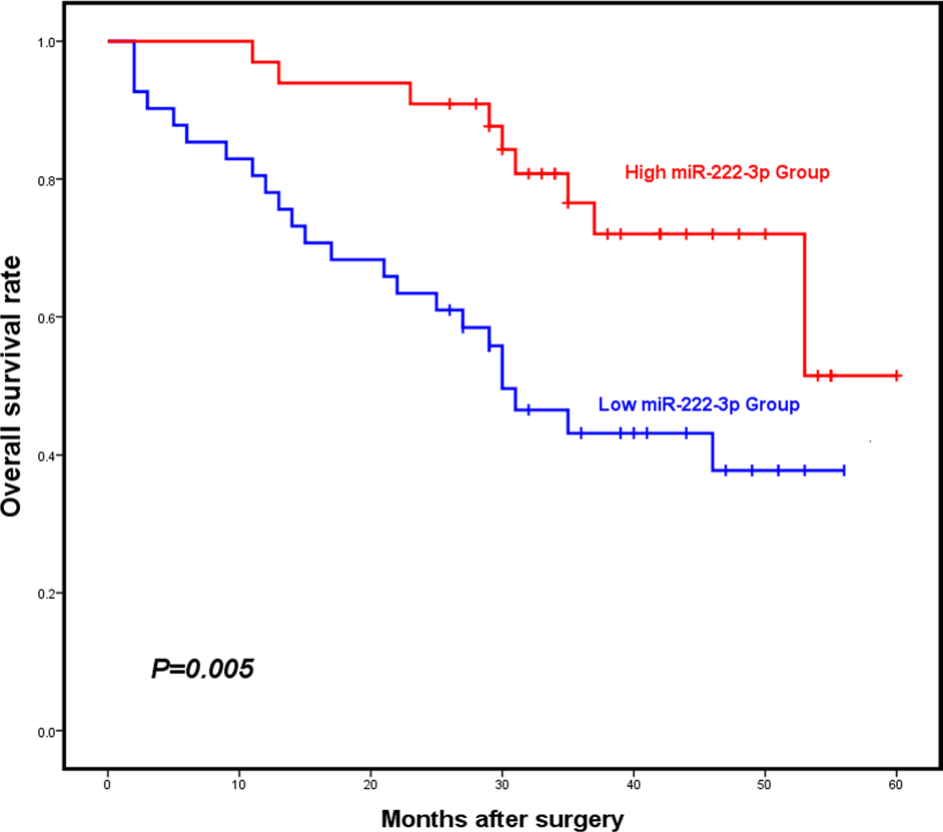
Elevated expression of miR-222-3p is associated with improved overall survival of EOC patients Kaplan-Meier overall survival curves for EOC patients with high and low miR-222-3p expression. EOC patients with high miR-222-3p expression (*N* = 33) had significantly longer overall survival than those with low miR-222-3p expression (*N* = 41) did (The mean overall survival time was 49.394 months *vs.* 33.435 months, *P* = 0.005**). All values were Mean ± SD, *P < 0.05, **P < 0.01, ***P < 0.001.

To determine whether miR-222-3p might be differentially expressed and associated with clinical outcome in patients with ovarian cancer we analyzed miR-222-3p expression by qRT-PCR in seventy-four EOC patients diagnosed at Xiangya Hospital of Central South University between September 2010 and December 2012. Patient characteristics are shown in Table 1. The relative expression levels of miR-222-3p according to different clinicopathologic factors are shown in Table 2. Decreased miR-222-3p expression was found to be significantly associated with histologic grade (Grade 1+2 *vs.* Grade 3: 73.97 *vs.* 23.49, *P* = 0.036). However, no significant correlation was observed between miR-222-3p and other clinicopathologic variables such as age, histologic type, and FIGO stage (all *P* > 0.05). Then the seventy-four cases were divided into two groups according to the relative expression levels of miR-222-3p (cutoff value = 1.60): 1) *high*, those who exhibited expression above 1.60; and 2) *low,* for those below 1.60. The relationships between miR-222-3p expression levels and different clinicopathologic factors are summarized in Table 3. But we did not observe any significant correlations between miR-222-3p expression and these clinicopathologic factors such as age, histologic type, FIGO stage, histologic differenciation, or histologic grade (all *P* > 0.05). Kaplan-Meier curves and survival curves showed that patients with high levels of miR-222-3p survived significantly longer than did the low-expressing group (the mean overall survival time was 49.394 months *vs.* 33.435 months; *P* = 0.005; Figure 1). Collectively, these results suggest a predictive role for miR-222-3p in the prognosis of EOC patients; that is, the higher the mean expression level of miR-222-3p, the longer the median overall survival time of EOC patients.

**Table 1:** Clinical characteristics of the cohort

Characteristics	EOC Cases	
	*N*	%
**EOC, *n***	74	100%
**Age (year)**		
< 50	37	50.00%
≥ 50	37	50.00%
Histologic type		
SC^1^	34	45.95%
Nonserous	40	54.05%
**FIGO^2^ stage**		
I	14	18.92%
II	19	25.68%
III	29	39.19%
IV	12	16.22%
**FIGO stage**		
I-II	33	44.59%
III-IV	41	55.41%
**Histologic differenciation**		
Well	1	1.35%
Well-moderate	9	12.16%
Moderate	17	22.97%
Moderate-poorly	11	14.86%
Poorly	36	48.65%
**Histologic grade**		
Grade 1	10	13.51%
Grade 2	27	36.49%
Grade 3	37	50.00%
**Recurrent disease at time of analysis**		
Yes	40	54.05%
No	34	45.95%
**Survival status at time of analysis**		
Dead	33	44.59%
Alive	41	55.41%

1 SC, Serous Cancer.

2 FIGO, International Federation of Obstetricians and Gynecologists.

**Table 2:** miR-222-3p relative expression levels according to different clincopathological characteristics of EOC patients

Characteristics	*n*	Relative miR-222-3p mRNA expression level^3^	*P* value^4^
**Age (years)**			
< 50	37	47.43 (0.3390–2.7926)	0.639
≥ 50	37	36.39 (0.5968–11.1916)	
**Histologic type**			
SC^1^	34	44.04 (0.2430–7.0423)	0.867
Nonserous	40	40.09 (0.5030–4.1786)	
**FIGO^2^ Stage at diagnosis**			
I-II	33	35.84 (0.5143–3.9281)	0.643
III-IV	41	46.80 (0.3294–9.9733)	
**Histologic grade at diagnosis**			
Grade 1+2	37	73.97 (0.5300–9.4022)	0.036*
Grade 3	37	23.49 (0.4234–4.3420)	

1 SC, Serous Cancer.

2 FIGO, International Federation of Obstetricians and Gynecologists.

3 Relative miR-222-3p expression (Median values) with 25th–75th percentile in parentheses.

4 P values ≤ 0.05 were considered significant according to the two-sample Student's t test; The P values represent significant differences between groups according to clinicopathological characteristics for miR-222-3p, respectively.

**Table 3:** Association of miR-222-3p expression with clinicopathological characteristics of EOC patients

Characteristics	Cases		miR-222 mRNA expression level^3^		*P* value^4^
	*N*	%	Low	High	
**EOC, *n***	74	100%	41	33	
**Age(year)**					
< 50	37	50.00%	22	15	0.363
≥ 50	37	50.00%	19	18	
**Histologic type**					
SC^1^	34	45.95%	18	16	0.129
Nonserous	40	54.05%	23	17	
**FIGO^2^ stage**					
I	14	18.92%	8	6	0.837
II	19	25.68%	10	9	
III	29	39.19%	15	14	
IV	12	16.22%	8	4	
**Histologic differenciation**					
Well	1	1.35%	0	1	0.079
Well-moderate	9	12.16%	3	6	
Moderate	17	22.97%	13	4	
Moderate-poorly	11	14.86%	8	3	
Poorly	36	48.65%	17	19	
**Histologic grade**					
Grade 1+2	37	50.00%	24	13	0.102
Grade 3	37	50.00%	17	20	

1 SC, Serous Cancer.

2 FIGO, International Federation of Obstetricians and Gynecologists.

3 The relative expression levels of miR-222-3p (cutoff value = 1.60): 1) high, those who exhibited expression above 1.60; and 2) low, for those below 1.60.

10 *P* values ≤ 0.05 were considered significant according to the two-sample Student's *t* test. The *P* values represent significant differences between groups according to clinicopathological characteristics for miR-222-3p, respectively.

A Cox proportional hazards analysis was used to further evaluate the potential of miR-222-3p expression as a prognostic biomarker (Table 4). Univariate survival analyses indicated that miR-222-3p expression (*P* = 0.010), histologic type (*P* = 0.019), histologic grade (*P* = 0.039) were associated with overall survival, while age (*P* = 0.247) and FIGO stage (*P* = 0.137) were not associated with overall survival. In the multivariate Cox proportional hazards analysis, which included miR-222-3p expression, histologic type, and histologic grade, miR-222-3p expression was found to be an independent prognostic factor for overall survival (*P* = 0.006; hazard ratio 0.347; 95% CI 0.164 to 0.734). The significant association of higher levels of miR-222-3p with good overall survival agrees with Figure 1.

**Table 4:** Univariate and multivariate analyses for overall survival of 74 EOC patients

Characteristics	Univariate analysis			Multivariate analysis		
	HR^1^	95% CI^2^	*P* value	HR^1^	95% CI^2^	*P* value
**Expression of miR-222-3p**						
(low vs. high)	0.371	0.175–0.786	0.010*	0.347	0.164–0.734	0.006**
**Age**						
(< 50 vs. ≥ 50)	1.512	0.751–3.045	0.247			
**Histologic type**						
(SC vs. Nonserous)	2.402	1.155–4.996	0.019*	1.931	0.887–4.202	**0.097**
**FIGO stage**						
( I–II vs. III–IV)	1.733	0.840–3.578	0.137			
**Histologic grade**						
(Grade 1+2 vs. Grade 3)	2.318	1.042–5.154	0.039*	0.114	0.848–4.692	**0.114**

1 HR: Hazard ratio;

2 CI: Confidence interval;

The bold number represents the *P*-values with significant differences.

### Negative correlation between miR-222-3p expression and mouse tumor size

**Figure 2:**
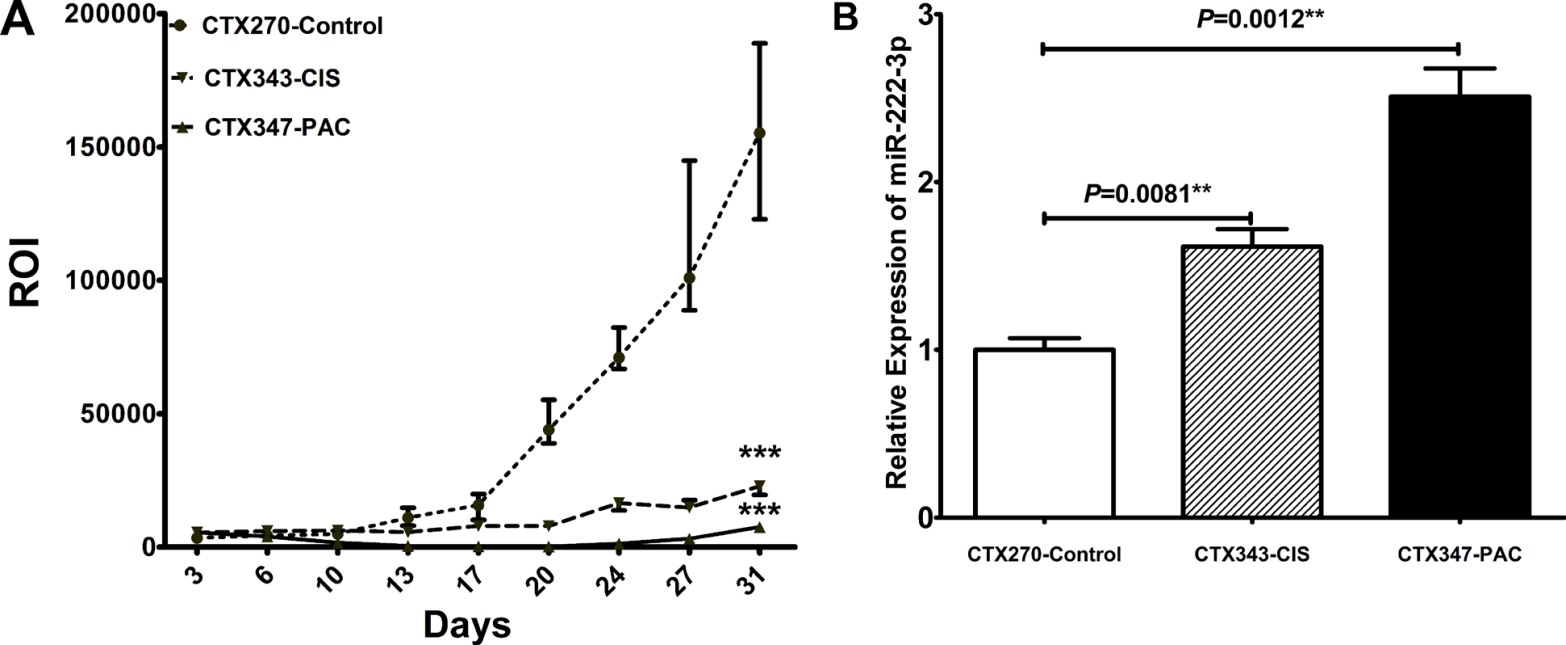
MiR-222-3p expression and tumor growth in different EOC athymic nude mouse models (**A**) The ROI area of tumors in nude mice. CTX343-CIS and CTX347-PAC nude mice received intraperitoneal injection of Cisplatin (5 mg/kg, weekly) and Paclitaxel (12 mg/kg, q3d) respectively. The CTX270-control mice received 0.9 % sodium chloride. (**B**) The mRNA levels of miR-222-3p in the CTX343-CIS, CTX347-PAC, and CTX270-control mice. All values were Mean ± SD, *P < 0.05, **P < 0.01, ***P < 0.001.

Next, we detected miR-222-3p expression levels in a mouse model of EOC. These mice included a group of untreated negative control mice designed as CTX270-control mice, and two groups of animals treated with either cisplatin (CTX343-CIS) or paclitaxel (CTX347-PAC). As shown in Figure 2A, quantification of tumor growth determined by the regions of interest (ROI) in the CTX343-CIS (*P* < 0.0001) and CTX347-PAC (*P* < 0.0001) mice showed a progressive decrease compared to the CTX270-control group. Quantification of miR-222-3p expression levels by qRT-PCR in the CTX343-CIS (*P* = 0.0081) and CTX347-PAC (*P* = 0.0012) mice revealed significant higher levels than those observed in the CTX270-control group (Figure 2B). These data showed a negative association between tumor growth after chemo-treatment and miR-222-3p expression levels.

### Correlation between miR-222-3p expression, proliferation and migration of human EOC cell lines

**Figure 3:**
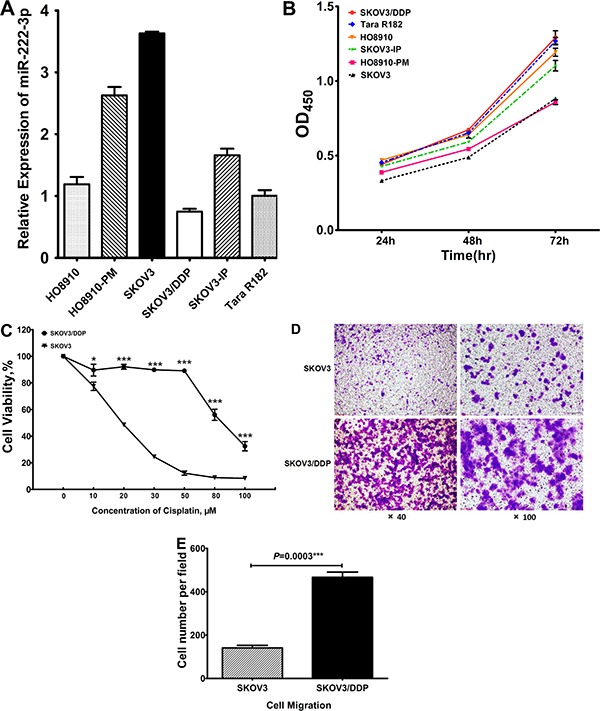
Contrary relationship between expression of miR-222-3p and proliferation, migration of human EOC cell lines (**A**) Differential relative mRNA expression of miR-222-3p in six EOC cell lines. (**B**) Cell growth analysis of these six EOC cell lines. (**C**) The cell viability of SKOV3 and SKOV3/DDP cells treated with cisplatin. (**D**–**E**) The basal cell migration ability of SKOV3 and SKOV3/DDP cells. Representative images were shown with the quantification of five randomly selected fields. All values were Mean ± SD, *P < 0.05, **P < 0.01, ***P < 0.001.

Our next objective was to investigate the role of miR-222-3p as a tumor suppressor-miR. First, we determined miR-222-3p expression by qRT-PCR analysis in six ovarian cancer cell lines (Tara R182, SKOV3, SKOV3/DDP, SKOV3-IP, HO8910 and HO8910-PM). As shown in Figure 3A, SKOV3, HO8910PM and SKOV3-IP had higher levels of miR-222-3p, while HO8910, Tara R182 and SKOV3/DDP had relatively low expression levels of miR-222-3p. Interestingly, we found an inverse correlation between the cell proliferation rates of these six cell lines and their miR-222-3p expression levels, suggesting a potential role for miR-222-3p in inhibiting cell growth and proliferation (Figure 3B). Thus, SKOV3/DDP and Tara R182 cells which exhibit high cell growth rate have low levels of miR-222-3p, while SKOV3 and HO8910-PM cells, which have a low cell growth rate, express high levels of miR-222-3p (Figure 3B).

Among these six cell lines, SKOV3/DDP is a cisplatin resistant cell line, while SKOV3 is cisplatin sensitive (*P* < 0.05; Figure 3C). miR-222-3p is highly expressed in the chemo-sensitive SKOV3 cells, but low in the chemoresistant SKOV3/DDP cells which correlates with our human data showing that patients that respond to chemotherapy have higher levels of miR-222-3p (Figure 1). Furthermore, these miR-222-3p low, chemoresistant SKOV3/DDP cells also are characterized by high migration capacity as determined by a transwell migration assay (*P* = 0.0003; Figure 3D, 3E). Similarly, the miR-222-3p high, chemosensitive SKOV3 cells have low migration capacity ( *P* = 0.0003; Figure 3D, 3E).

### MiR-222-3p overexpression decreases EOC cell proliferation and migration *in vitro*

**Figure 4:**
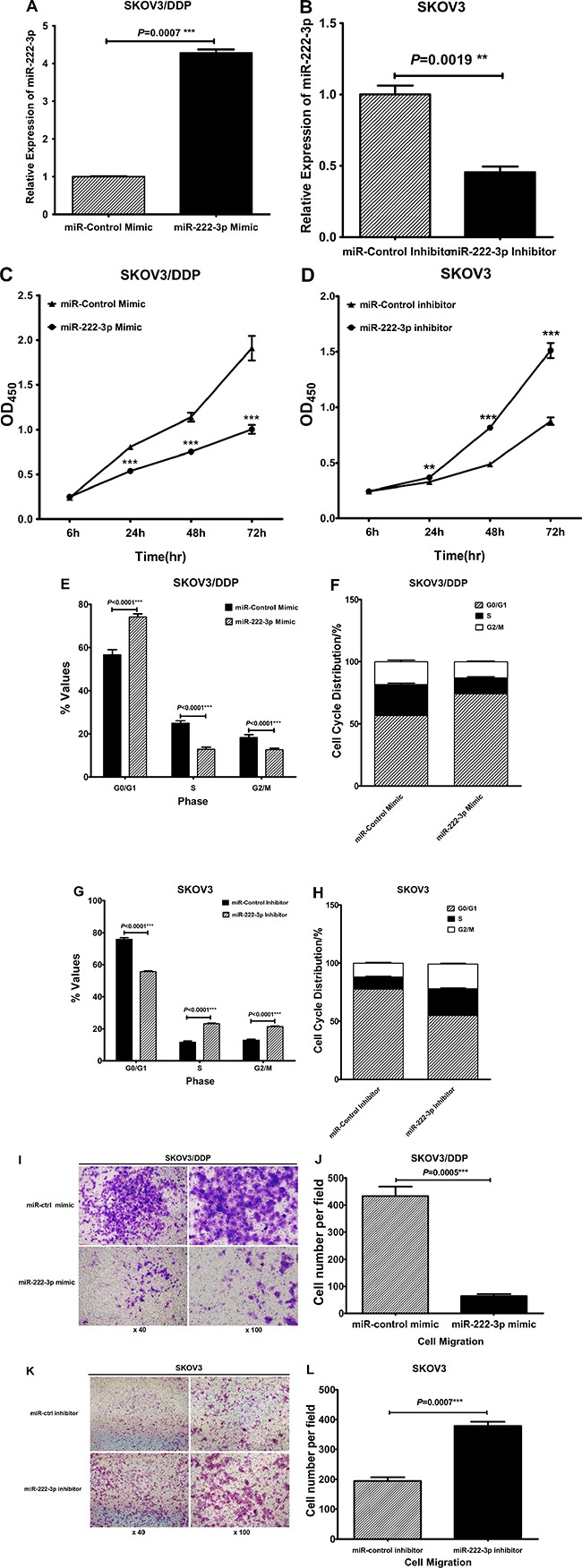
MiR-222-3p overexpression decreases EOC cell proliferation and migration *in vitro* (**A**) Transfection efficiency of miR-222-3p mimic in SKOV3/DDP by qRT-PCR 48h after transfection, using miR-control mimic as a negative control. (**B**) Transfection efficiency of miR-222-3p inhibitor in SKOV3 by qRT-PCR 48 h after transfection, using miR-control inhibitor as a negative control. (**C**) Cell proliferation was analyzed using a CCK-8 assay. The proliferation of SKOV3/DDP transfected with miR-222-3p mimic was reduced, compared with that transfected with miR-control mimic. (**D**) Cell proliferation was analyzed using a CCK-8 assay. The proliferation of SKOV3 transfected with miR-222-3p inhibitor was reduced, compared with that transfected with miR-control inhibitor. (**E**–**F**) Effects of miR-222-3p mimic on SKOV3/DDP cell cycle distribution. (**G**–**H**) Effects of miR-222-3p inhibitor on SKOV3 cell cycle distribution. (**I**–**J**) Transwell migration assay of SKOV3/DDP transfected with miR-222-3p mimic or miR-control mimic. Representative images were shown with the quantification of five randomly selected fields. (**K**–**L**) Transwell migration assay of SKOV3 transfected with miR-222-3p inhibitor or miR-control inhibitor. Representative images were shown with the quantification of five randomly selected fields. All values were Mean ± SD, *P < 0.05, **P < 0.01, ***P < 0.001.

To further explore the potential role of miR-222-3p in EOC cells, we transiently transfected miR222-3p mimic into SKOV3/DDP and HO8910 cell lines (low expression of miR-222-3p). Control cells were transfected with miR-control mimic as negative control (NC). Transfection efficiency was confirmed by qRT-PCR (*P* = 0.0007, Figure 4A; *P* < 0.0001, Supplementary Figure S1A–S1H). Additionally, we also transiently transfected miR-222-3p inhibitor into SKOV3 and HO8910-PM cell lines (high expression of miR-222-3p). Control cells were transfected with miR-control inhibitor as negative control (NC). Transfection efficiency was also detected by qRT-PCR (*P* = 0.0019, Figure 4B; *P* < 0.0001, Supplementary Figure S1B).

We next performed a CCK-8 cell proliferation assay and data showed that the proliferation rates for SKOV3/DDP and HO8910 cells transfected with the miR222-3p mimic were lower than for cells transfected with the miRcontrol mimic (*P* < 0.0001, Figure 4C; *P* < 0.0001, Supplementary Figure S1C); In contrast, after transfecting miR-222-3p inhibitor into SKOV3 and HO8910-PM, the cell proliferation rate was increased compared with the control cells (*P* < 0.0001, Figure 4D; *P* < 0.0001, supplementary Figure S1D). A symbolic characteristic of cancer cells is a proliferation advantage over normal cells resulting from an impaired regulation of cell cycle [23, 24]. Therefore, the effect of miR-222-3p on EOC cell cycles was investigated using flow cytometry. After transfecting miR-222-3p mimic into SKOV3/DDP, the percentage of cells in G0/G1 phase increased and that of S and G2/M phases were decreased compared to the control cells (*P* < 0.0001, Figure 4E, 4F, Supplementary Figure S1E and Supplementary Figure S1F). On the contrary, if we transfect miR-222-3p inhibitor into SKOV3, we observed an increase in cell proliferation demonstrated by a lower G0/G1 phase cell percentage and a higher percentages of cells in S and G2/M phases compared to the control cells (*P* < 0.0001; Figure 4G, 4H, Supplementary Figure S1G, and S1H).

Furthermore, overexpression of miR-222-3p in SKOV3/DDP cells would block cellular migration (*P* = 0.0005; Figure 4I, 4J), while inhibition of miR-222-3p in SKOV3 cells would promote cellular migration (*P* = 0.0007; Figure 4K, 4L).

### MiR-222-3p overexpression reduces ovarian cancer cell proliferation by inhibiting phosphorylation of AKT

**Figure 5:**
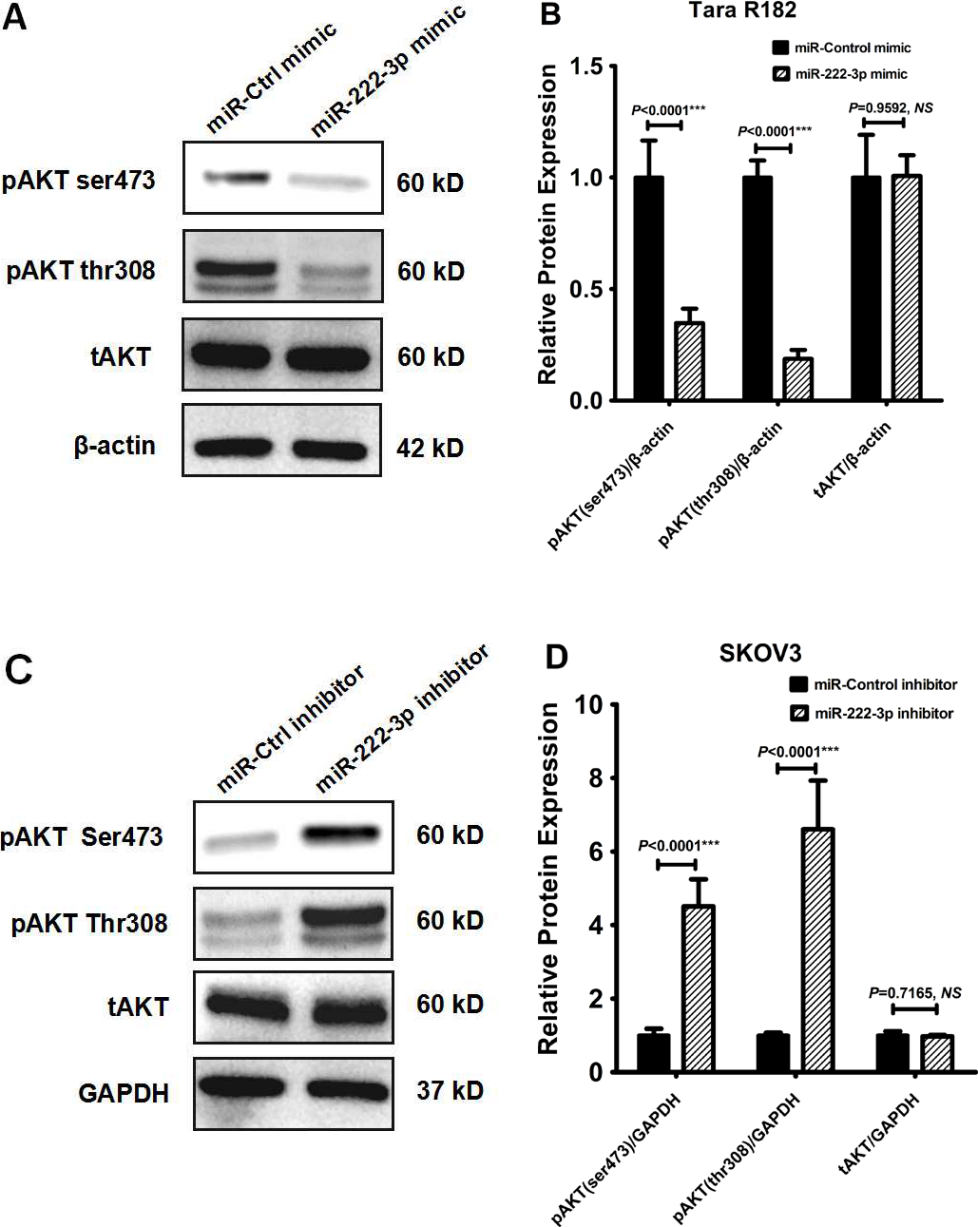
MiR-222-3p overexpression reduces ovarian cancer cell proliferation by inhibiting phosphorylation of AKT (**A**) Tara R182 cell line was transfected with miR-222-3p mimic, Western blot analysis of AKT phosphorylation levels at both Ser473- and Thr308- residues and total AKT were detected. Expression of β-actin was used as a loading control. (**B**) The relative expressions of GNAI2, pAKT (ser473), pAKT (thr308) and total AKT proteins were normalized to β-actin. (**C**) SKOV3 cells were transfected with miR-222-3p inhibitor, Western blot analysis of AKT phosphorylation levels at both Ser473- and Thr308- residues and total AKT were detected. Expression of GAPDH was used as a loading control. (**D**) The relative expressions of GNAI2, pAKT (ser473), pAKT (thr308) and total AKT proteins were normalized to GAPDH.

It has been shown that the phosphorylation status of AKT is closely correlated with cellular proliferation, thus, we tested the hypothesis that miR-222-3p could regulate cell proliferation by affecting the AKT pathway. Our data showed that AKT phosphorylation at both Ser473- and Thr308- residues was inhibited after transfection of miR-222-3p mimic into Tara R182 cells (both *P* < 0.0001; Figure 5A, 5B); this effect was time dependent reaching maximal inhibition at 72 h (supplementary Figure S2). miR-221, a member of the same cluster had similar inhibitory effect on AKT phosphorylation levels (supplementary Figure S2). Moreover also we inhibited miR-222-3p expression using its inhibitor, we found that AKT phosphorylation at both residues was increased in SKOV3 cells, comparing to the control cells (both *P* < 0.0001; Figure 5C, 5D). However, the observed decrease/increase of pAKT after transfection are not due to total AKT (tAKT) differences since we did not found changes on the expression levels of tAKT following miR-222-3p mimic/inhibitor transfection (both *P* > 0.05; Figure 5A–5D and Supplementary Figure S2). Thus, our results suggest that the target of miR-222-3p is not AKT but potentially a protein regulator of AKT-phosphorylation.

### MiR-222-3p inhibits GNAI2 expression

**Figure 6:**
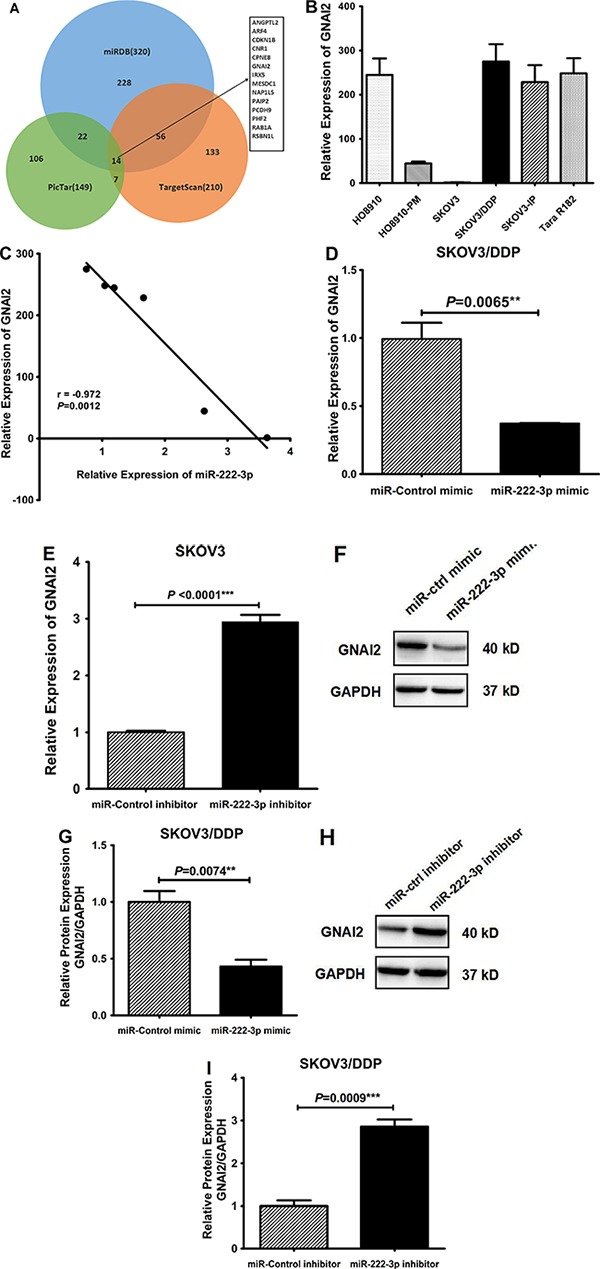
miR-222-3p down-regulates GNAI2 mRNA and protein levels (**A**) Schematic diagram of the predicted miR-222-3p-targeting genes by TargetScan, PicTar and miRDB. There are fourteen miRNAs shared by three software program results (each circle and the number in brackets represent the corresponding software and its predicting gene number). (**B**) Differential relative mRNA expression of GNAI2 in these six EOC cell lines. (**C**) The Pearson's Correlation analysis clearly showed negative correlation between miR-222-3p and GNAI2 mRNA expression in these EOC cell lines (*r* = −0.972, *P* = 0.0012). (**D**) Relative expression of GNAI2 mRNA in SKOV3/DDP transfected with miR-222-3p mimic or miR-control mimic. (**E**) Relative expression of GNAI2 mRNA in SKOV3 transfected with miR-222-3p inhibitor or miR-control inhibitor. (**F**–**G**) Western blot assay of the GNAI2 protein levels in SKOV3/DDP transfected with miR-222-3p mimic or miR-control mimic, and the relative expression of GNAI2 was normalized to GAPDH (G). (**H**–**I**) Western blot assay of the GNAI2 protein levels in SKOV3/DDP transfected with miR-222-3p inhibitor or miR-control inhibitor, and the relative expression of GNAI2 was normalized to GAPDH (I). All values were Mean ± SD, *P < 0.05, **P < 0.01, ***P < 0.001.

To identify the potential intermediary responsible for pAKT inhibition induced by miR-222-3p, firstly we performed an online bioinformatics prediction search for putative mRNA targets of miR-222-3p by using three online predicting algorithms (PicTar, TargetScan and miRDB). And then fourteen candidate genes were commonly predicted to be the possible targets of miR-222-3p by all of the three algorithms (Figure 6A); finally, we evaluated whether these predicted target genes would be down-regulated *in vitro* after overexpression of miR-222-3p in ovarian cancer cells using qRT-PCR. Among the down-regulated candidates (data not shown), only GNAI2 has been previously reported to be an activator of AKT [25]; therefore, we evaluated GNAI2 mRNA expressions in the six ovarian cancer cell lines by qRT-PCR (Figure 6B). We observed a negative correlation between miR-222-3p and GNAI2 expression levels (*r* = −0.972, *P* = 0.0012; Figure 6C). Furthermore in SKOV3/DDP cells, GNAI2 was suppressed following overexpression of miR-222-3p (*P* = 0.0065; Figure 6D) and in SKOV3 (high miR-222 and low GNAI2) GNAI2 was increased when miR-222-3p was inhibited by its inhibitor (*P* < 0.0001; Figure 6E). We also repeated this in HO8910 and HO8910-PM cells and achieved similar results (all *P* < 0.0001; supplementary Figure S3A, S3B). Western blot analysis further confirmed the findings that described at the mRNA level at the protein level in SKOV3/DDP and Tara R182 cell lines (all *P* < 0.05; Figure 6F–6I, Supplementary Figure S3C–S3F).

### MiR-222-3p directly targets the GNAI2 3′-UTR

**Figure 7:**
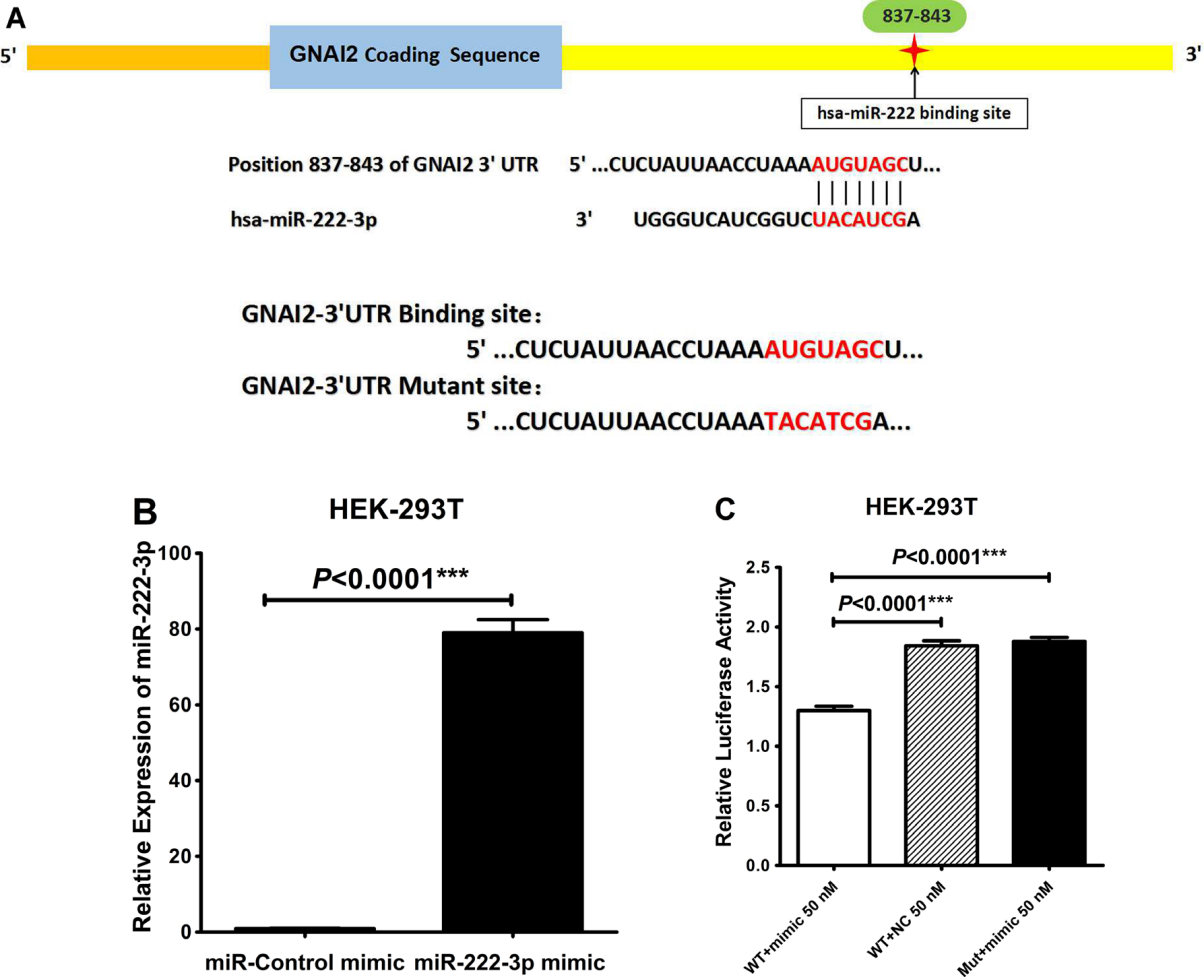
miR-222-3p directly targets the 3'UTR of GNAI2 (**A**) Schematic diagram of the putative binding site in GNAI2 mRNA 3'UTR for miR-222-3p (The identical GNAI2 wild-type (WT) seed sequences AUGUAGC and mutant (Mut) 3'UTR sequences TACATCG for miR-221/222 as shown above). (**B**) Transfection efficiency of miR-222-3p mimic and miR-control mimic in HEK-293T by qRT-PCR. (**C**) Relative luciferase activity in HEK-293T cells co-transfected the luciferase-3'UTR construct (wild or mutant type) with miR-222-3p mimic, and the ratio of *Renilla*/Firefly luciferase activities was determined. All values were Mean ± SD, *P < 0.05, **P < 0.01, ***P < 0.001.

To verify that GNAI2 is a direct target of miR-222-3p the full-length 3′-UTR fragment containing the predicted miR-222-3p binding site (positions 837–843) was cloned downstream of the luciferase open reading frame of a psi-CHECK2™ vector. We also constructed a luciferase reporter containing a mutant version generated by mutating the predicted seed sequenced (Figure 7A). Before co-transfection, the transfection efficiency of miR-222-3p mimic was confirmed by qRT-PCR in HEK-293T cells (*P* < 0.0001, Figure 7B), using miR-control mimic as negative control (NC). We then co-transfected the luciferase-3’-UTR construct (wild or mutant type) with miR-222-3p mimic into HEK-293T cells, and the ratio of *Renilla/Firefly* luciferase activities was determined. Relative to the miR-control mimic, miR-222-3p mimic reduced the luciferase ratio of the wild-type 3’-UTR construct, but not that of the mutant (*P* < 0.0001; Figure 7C). Collectively, these results demonstrated that GNAI2 is a direct target gene of miR-222-3p.

### MiR-222-3p suppresses EOC cell proliferation via the GNAI2/AKT pathway

**Figure 8:**
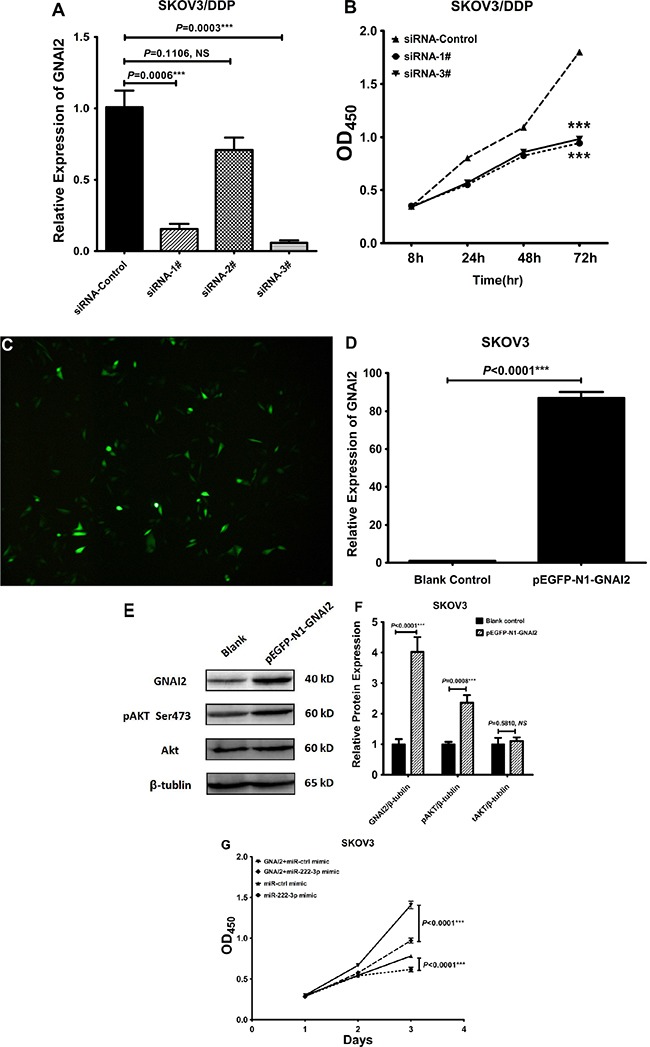
MiR-222-3p suppresses EOC cell proliferation via GNAI2/AKT pathway (**A**) GNAI2 expression in SKOV3/DDP cells transfected with siGNAI2-1#, 2#, 3# and siRNA-control. Changes in mRNA abundance were determined by qRT-PCR. (**B**) Cell proliferation was analyzed using a CCK-8 assay. The proliferation of SKOV3/DDP transfected with siGNAI2-1# and -3# was reduced, compared with that transfected with siRNA-control. (**C**) Green fluorescence protein expression in SKOV3 cells transfected with pEGFP-N1-GNAI2 plasmid (500 ng) was observed by fluorescence microscope (Magnification, **×** 100) 48 h after transfection. (**D**) Change in mRNA abundance was determined by qRT-PCR 48h post pEGFP-N1-GNAI2 transfection in SKOV3 cells. (**E**–**F**) Western Blot analysis of GNAI2, pAKT (ser473), and AKT 48h post pEGFP-N1-GNAI2 transfection in SKOV3 cells, and the relative expressions of GNAI2, pAKT (ser473), and total AKT proteins were normalized to β-actin (F). (**G**) Co-transfection of pEGFP-N1-GNAI2 and miR-222-3p mimic or miR-control mimic into SKOV3. The cell proliferation rates were analyzed by CCK-8 assay. All values were Mean ± SD, *P < 0.05, **P < 0.01, ***P < 0.001.

Our next objective was to investigate how GNAI2 function in EOC cells. Thus, we used SKOV3/DDP cell lines showing high levels of GNAI2, and transfected them with three siRNAs to specifically silence GNAI2 expression (described in the Methods section). The transfection efficiency was examined by qRT-PCR, using a scrambled RNA sequence as a negative control (siRNA-control). Two siRNAs, siGNAI2-1# (*P* = 0.0006) and -3# (*P* = 0.0003) were effective in inhibiting GNAI2 and we used these in further experiments (Figure 8A). By using the CCK8 assay we found that the cellular proliferation rate of SKOV3/DDP was reduced when transfected with siGNAI2-1# and -3# (both *P* < 0.0001; Figure 8B). But the cellular migration of SKOV3/DDP cells transfected with siGNAI2-1# and -3# showed no significant differences, comparing to the control cells (both *P* > 0.05; Figure not shown).

To further confirm that GNAI2 was the intermediary factor mediating miR-222-3p inhibition of AKT phosphorylation and EOC cells proliferation, a recombinant pEGFP-N1 plasmid containing a full-length GNAI2 ORF (Open Reading Frame) without 3′-UTR was constructed and transiently transfected into SKOV3, which expressed low levels of GNAI2. We analyzed the transfection efficiency by fluorescence microscopic and qRT-PCR in SKOV3 cells (Figure 8C, 8D) and next we detected the expression levels of GNAI2, pAKT and tAKT protein by Western blot analysis. Cells transfected with pEGFP-N1-GNAI2 showed increased GNAI2 and pAKT compared with the control group with no changes in tAKT (Figure 8E, 8F). And by CCK-8 assay, we further confirmed that GNAI2 overexpression enhanced cells proliferation while the miR-222-3p mimic suppressed it (Figure 8G).

### Inverse correlation between miR-222-3p and GNAI2 expression in ovarian cancer patient samples

**Figure 9:**
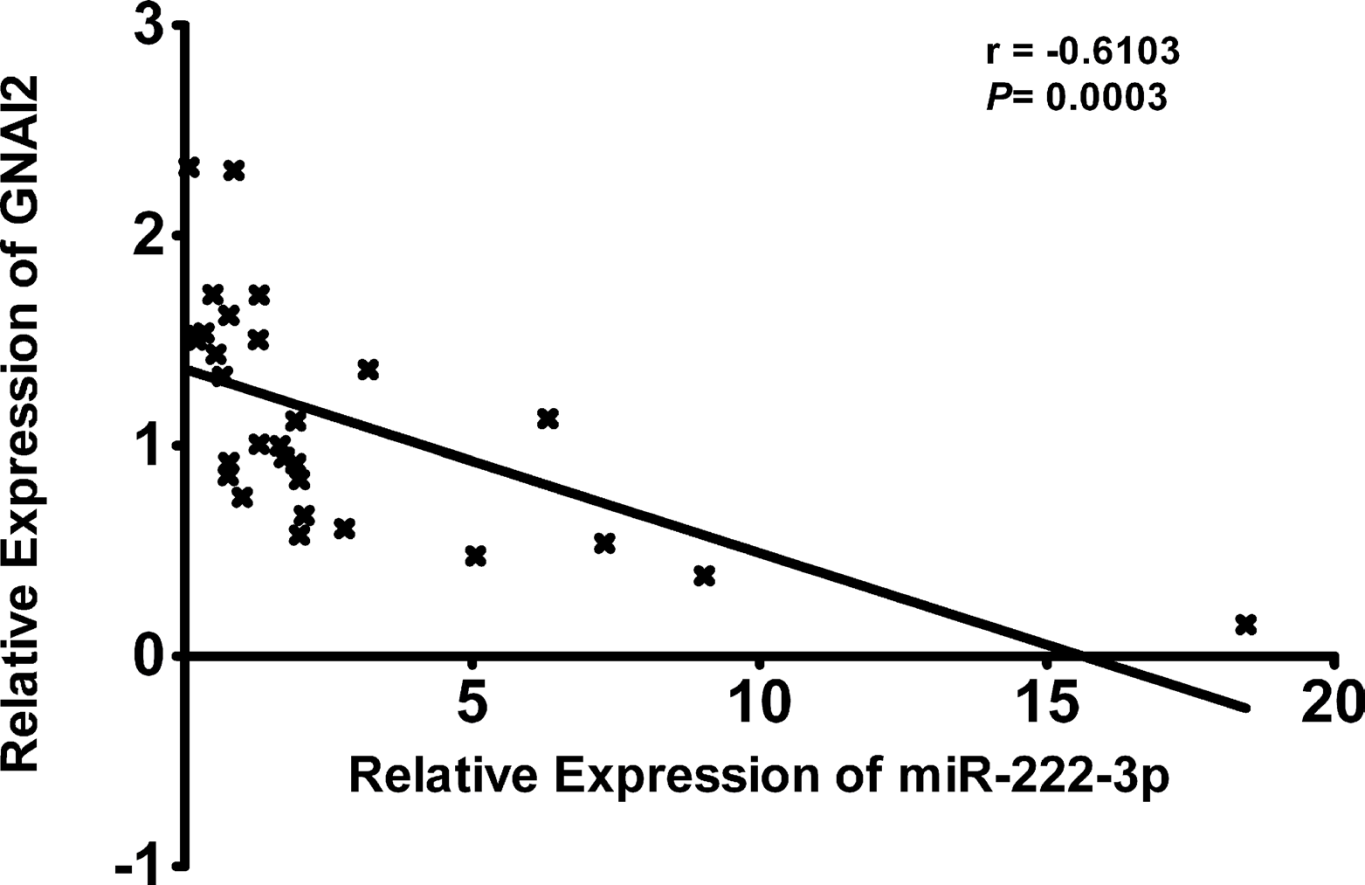
The Pearson's Correlation analysis clearly showed negative correlation between miR-222-3p and GNAI2 mRNA expression in tumor samples from EOC patients (n = 30, r = −0.6103, P = 0.0003)

Using twenty EOC patients’ samples and ten normal ovary samples we examined miR-222-3p and GNAI2 expression by qRT-PCR. A Pearson correlational analysis of these thirty samples exhibited an inverse correlation between GNAI2 and miR-222-3p expression levels (*r* = −0.6103, *P* = 0.0003; Figure 9), which was consistent with the results found in the cell lines (*r* = −0.972, *P* = 0.0012; Figure 6C). Collectively, we demonstrated that GNAI2 mRNA expression was inversely correlated with the expression of miR-222-3p, suggesting that miR-222-3p could negatively regulate GNAI2 expression in human EOC.

## DISCUSSION

In the present study we identify a novel target for miR-222-3p associated with AKT regulation and function. We described GNAI2 as a direct target of miR-222-3p that controls cell proliferation in ovarian cancer cells. Furthermore, our data showed a direct correlation between miR-222-3p expression and overall survival in ovarian cancer patients.

According to Cancer Statistics in China, 2015, there is an upward trend in mortality of women due to gynecological cancers, including ovarian cancer [26]. Ovarian cancer also ranks fifth in cancer related-deaths among American women [27]. The pathogenesis of OC is concealed, and because of the lack of any effective screening methods to detect the tumors at early stages, the majority of patients are diagnosed at Stage III, some even at Stage IV [28, 29]. Although 80–90% of patients initially respond to chemotherapy, most eventually recur and become chemoresistant [3, 4]. As such, recurrence, chemoresistance and metastasis are the main reasons of mortality in ovarian cancer.

As stated above, miR-222 plays multiple roles in many cancer types. Initially, miR-222 was described as an oncogenic microRNA in gastric cancer [30], cervical cancer [13], bladder cancer [31], hepatocellular carcinoma [32], breast cancer [33], endometrial carcinoma [34], melanoma [35, 36], pancreatic cancer [37], glioblastoma [38], thyroid cancer [39], multiple myeloma [40, 41], chronic lymphocytic leukemia [42], and oral squamous cell carcinoma [43]. However, in 2005, Felli's group [16] discovered that KIT, a well-known oncogene, is targeted by miR-221/222 in erythroblastic leukemia, thus illustrating miR-221/222's function as a tumor suppressor in human erythroblast cells. Analogously, in gastrointestinal stromal tumors, Wiemer *et al.* [17] found in 2013 that overexpressing miR-222 significantly inhibits cellular proliferation, affects cell cycle kinetics and induces apoptosis by down-regulating its predicted target genes KIT and ETV1 in human gastrointestinal stromal tumors. Later, in 2015, Ihle *et al.* [18] further proved that miRNA-222 can induce apoptosis *via* a signaling cascade involving KIT, AKT and BCL2. In prostate cancer, Seki *et al.* [44, 45] revealed that miR-222 was able to directly target Ecm29 so as to significantly suppress cancer cell migration and invasion, but many other researchers have reported opposing results where miR-222 promoted prostate cancer cell proliferation or migration [14, 46]. Interestingly, one group announced that miR-222 promoted growth in H460, which is a human non-small cell lung cancer cell line [47]; however, almost simultaneously, Yamashita and associates [48], using six lung cancer cell lines, demonstrated that miR-222 promoted growth in two cell lines but suppressed growth in three lung cancer cells. Therefore, whether miR-222 functions as an onco-miR or a tumor suppressor-miR mainly depends upon the cellular context of cancer cells and their target genes.

In the current study we present several findings. First, by measuring miR-222-3p expression in 74 EOC patients, we found that patients with high levels of miR-222-3p survived significantly longer than did the low expressing group, which indicated that miR-222-3p might constitute a better prognostic index for EOC patients. In addition, our *in-vivo* experiments showed that chemo-sensitive tumors in the mice expressed relatively higher levels of miR-222-3p, supporting the clinical data. Therefore, to assess the functional role of miR-222-3p in EOC, we first investigated the basal expression levels of miR-222-3p in six EOC cell lines. In order to explore the effects of miR-222-3p on EOC cell proliferation, we transiently transfected miR222-3p mimic into the low-expressing miR-222-3p cell lines, and found that the cellular proliferation was inhibited significantly, which correlated with changes in the phosphorylation state of AKT. Western blot analysis showed a reduction in pAKT levels (ser473, thr308), but no change in total AKT expression, suggesting that the target gene for miR-222-3p was not AKT expression. Indeed, gene analysis identified GNAI2 as the potential target of miR-222-3p for regulating pAKT.

Heterotrimeric guanine nucleotide-binding proteins (G proteins) consist of three subunits:α, β and γ. According to Downes and Gautam [49], at least 21 α subunits, 6 β subunits and 12 γ subunits have now been identified, which can couple to G-protein-coupled receptors (GPCR), and then mediate a variety of biologic processes, including differentiation and development [50]. Based on the amino acid sequence identity of the Gα subunits, G proteins can be categorized into four distinct classes: Gs (stimulates adenylate cyclase [AC]), Gi (inhibits AC), Gq (activates phospholipase Cβ [PLCβ]), and G12/13 ( which trigger small Rho-GTPases) [51].

The inhibitory G alpha proteins (Giα proteins) attenuate intracellular cAMP expression by inhibiting AC, and therefore suppress CREB activity [52]. This group includes three polypeptides: GNAI1 (G protein alpha inhibiting activity polypeptide 1, Galphai1, Giα1), GNAI2 (G protein alpha inhibiting activity polypeptide 2, Galphai2, Giα2), and GNAI3 (G protein alpha inhibiting activity polypeptide 3, Galphai3, Giα3) [25]. Classically, Giα proteins transduce signals from Giα protein-coupled receptors (GiαPCRs) to their downstream cascades triggered by hormones or neurotransmitters [53, 54], and are thus involved in a plethora of physiologic and pathologic processes, including tumorigenesis. Giα proteins can activate the Ras-Raf-MEK-ERK mitogenic pathway by membrane recruitment of a novel isoform of Rapl GTPase-activating protein(rap1GAPII) and reduction of GTP-bound Rap1, thereby regulating cell growth [55]. Furthermore, Giα proteins differentially regulate the activation of AKT, mTORC1 (mammalian target of rapamycin complex 1) and ERK1/2 (extracellular signal-regulated kinases 1/2) by different families of growth factors in breast cancer [25]. As both PI3K/AKT/mTORC1 and ERK1/2 pathways are two important components in cellular growth and malignant transformation, Giα proteins should play essential roles in breast cancer progression, invasion and migration [25].

Our data demonstrates that miR-222-3p is a major regulator of GNAI2 expression and its function through its effect on the AKT pathway, a central regulator of cell proliferation and cell death. In conclusion, miR-222-3p might function as a tumor suppressor since it is negatively correlated with overall survival in EOC patients and chemo-response. Using mouse EOC models, we demonstrated an inverse relationship between miR-222-3p expression and mouse tumor size, and *in-vitro* experiments showed that its overexpression decreased cell proliferation, affected cell cycle kinetics and inhibited migration, thereby acting as a tumor suppressor. These findings suggest that miR-222-3p/GNAI2/AKT axis, might constitute a potential new therapeutic target for ovarian cancer.

## MATERIALS AND METHODS

### Patients and samples

This study was approved by the ethics committee of Xiangya Hospital (Central South University, Changsha, China). Written informed consent was obtained from all of the patients. All specimens were handled and made anonymous according to our investigational review board and ethics committee guidelines. Paraffin-embedded tissue samples from 74 patients with EOC were obtained from the pathology department of Xiangya Hospital between July 2010 and Dec 2012, of whom we had complete clinical and follow-up data. The follow-up periods were calculated from the date of surgery to death or last follow-up, and patients were excluded if they had incomplete medical records or inadequate follow-up. The median follow-up time was 30 months (range: 3 to 56 months). Median ages of EOC and benign ovarian tumor patients were 49 years, and ranged from 21 to 75 years. Detailed patient characteristics are summarized in Table 1. For quantitative reverse-transciptase polymerase chain reaction (qRT-PCR), We collected another 20 fresh ovarian tumor samples during surgical resection of the lesion between May 2012 and Nov 2014 at the obstetrics and gynecology department of Xiangya Hospital. We also obtained 10 normal ovarian tissue samples from patients with other diseases whose ovaries should be routinely removed. All collected-tissue samples were immediately snap-frozen using liquid nitrogen and then stored at −80°C. Histologic diagnosis and grading of tumors were carried out with FigO 2009* (FIGO Committee and Working Group Publications) by two pathologists.

### Mouse xenograft studies

The Yale University Institutional Animal Care and Use Committee approved all *in vivo* studies described. Athymic nude mice were randomly chosen and divided into three groups (three animals per condition) depended on different treatments. Intra-peritoneal (*i.p.*) tumors were established in all these athymic nude mice using 4 × 10^6^ mCherry + OCSC1-F2 ovarian cancer cells as previously described [56]. Injection of cancer cells is designated as day zero and treatment commenced between day three to five. Establishment of *i.p.* tumors was confirmed by live imaging (*In-Vivo* FX PRO, Bruker Corp., Billerica, MA) prior to treatment [94]. Paclitaxel was given at 12 mg/kg q3d and Cisplatin at 5 mg/kg weekly. All treatments were given by *i.p.* injection. Tumor growth was monitored q3d by live imaging and response to treatment was assessed using the ROI area as previously described [57, 58].

### Cell lines and regents

The epithelial ovarian cancer cell lines HO8910 (serous cystadenocarcinoma), HO8910-PM (highly invasive HO8910, serous cystadenocarcinoma) and SKOV3-IP (highly invasive SKOV3, serous papillary cystadenocarcinoma) were kindly provided by Professor Wu Xiaoying's laboratory (Xiangya Medical School, Central South University, Changsha, China). The epithelial ovarian cancer cell lines SKOV3 (serous papillary cystadenocarcinoma) and SKOV3/DDP (cisplatin-resistant SKOV3, serous papillary cystadenocarcinoma) were offered by Li Xiong (Xiangya Medical School, Central South University, Changsha, China). The epithelial ovarian cancer cell line Tara R182 was given by Professor Gil Mor (Yale University, New Haven, USA). HEK-293T and HO8910-PM cell lines were cultured in Dulbecco's modified Eagle's medium (DMEM) (Gibco, Carlsbad, CA), while the other ovarian cell lines (SKOV3, SKOV3/DDP, HO8910 and Tara R182) were maintained in RPMI-1640, supplemented with 10% fetal bovine serum (FBS) (Gibco, Carlsbad, CA), 1% nonessential amino acid (NEAA) (Gibco, Carlsbad, CA), 100 μg/ml penicillin (Sigma), and 100 μg/ml streptomycin (Sigma). All cells were cultured at 37°C in a humidified 5% CO_2_ incubator.

### Cell transfection

Between 5 **×** 10^4^ to 5 × 10^5^ cells/ml were plated without antibiotics for ~24 h prior to transfections. Transient transfections of the chemically synthesized miRNA mimic/inhibitor, siRNAs, scrambled controls (RiboBio, Guangzhou, China) and plasmids (GenePharma, Suzhou, China) were performed using Lipofectamine 2000 (Invitrogen, Carlsbad, CA, USA) at indicated concentrations according to the manufacturer's instructions once cells were at 75% – 85% confluence. For each well of a 6-well plate, cells were transfected with 50 nM of miRNA mimic/NC, 100 nM of miRNA inhibitor/NC, 50 nM of siRNA/NC or 500 to 1000 ng of recombinant plasmid (described below) using Lipofectamine 2000 (Invitrogen, Carlsbad, CA, USA). After six hours of transfection, the culture medium was replaced with fresh medium containing 10% FBS. All transfections were performed for 24 h or 48 h, prior to harvesting for the following assays.

The sequences of the above small molecules are as follows:

hsa-miR-222-3p mimic: agcuacaucuggcuacugggu; miRNA mimic NC: UUUGUACUACACAAAAGUA CUG.
hsa-miR-222-3p inhibitor: gcgauguagaccgaugacca; miRNA inhibitor NC: AAACAUGAUGUGUU UUCAUGAC.
Si-h-GNAI2_001: forward, 5′-CCAGCUACAUC CAGAGUAAdTdT-3′;
reverse, 3′-dTdT GGUCGAUGUAGGUCUC AUU-5′.
Si-h-GNAI2_002: forward, 5′-CUAAGAUGAU CGACAAGAAdTdT -3′;
reverse, 3′-dTdT GAUUCUACUAGCUGUUC UU-5′.
Si-h-GNAI2_003: forward, 5′-CAUCAUGGCCA UUGUCAAA dTdT -3′;
reverse, 3′-dTdT GUAGUACCGGUAACAGU UU-5′.

### RNA extraction, reverse transcription (RT)-PCR and quantitative real-time PCR

Total RNAs from cells or fresh tissues were extracted using TRIzol reagent (Invitrogen, CA). Reverse-transcribed complementary DNA was synthesized using the GoScript Reverse Transcription System (Promega, Madison, WI, USA). After diluting one part cDNA with four parts Nuclease-free water, real-time polymerase chain reaction (PCR) was then performed using the Applied Biosystems 7500 Real-Time PCR System and the GoTaq qPCR Master Mix (Promega, Madison, WI, USA) under the following conditions: predenaturation at 95°C for 10 minutes, followed by 40 cycles of denaturation at 95°C for 15 sec and annealing and extension at 60°C for 1 minute. The results were normalized to GAPDH expression.

The following primers were used:

GNAI2 forward, 5′-AAGTGGTTCACAGACA CGTCCATC-3′;
and reverse, 5′-GCGCTTATTCAGGTCCTCA AACTTA-3′;
GAPDH forward, 5′-GCACCGTCAAGGCTG AGAAC-3′;
and reverse, 5′-TGGTGAAGACGCCAGTGGA-3′.

For miRNA detection, the reverse transcribed cDNA was synthesized with the All-in-One™ miRNA First-Strand cDNA Synthesis Kit (GeneCopoeia, Rockville, MD, USA), and the relative miR-222-3p expression levels were normalized against U6 small nuclear RNA expression, using the All-in-One™ miRNA qRT-PCR Detection Kit (GeneCopoeia, Rockville, MD, USA) and their respective All-in-One™ miRNA qPCR Validation primer (GeneCopoeia, Rockville, MD, USA). The comparative CT method for relative quantification of gene expression (Applied Biosistems) was used to determine miRNA expression. Delta-delta-Cycle Threshold (ddCt) values were calculated relative to the average of the relevant normalization genes.

### Western Blot analysis

For Western Blot Analysis, the cells were lysed using the mammalian protein extraction reagent RIPA Lysis Buffer (Beyotime, Haimen, China) supplemented with a protease inhibitor cocktail (Roche, Mannheim, Germany) or 1%PMSF(Wuhan, China) and then clarified by centrifugation. Protein concentration was quantified using the BCA Protein Assay Kit (Pierce). The proteins (30 μg or 50 μg) were electrophoresed by sodium dodecyl sulfate–polyacrylamide gel electrophoresis (SDS-PAGE) and then transferred onto PVDF membranes. Immunoblotting was performed using the human anti-G protein alpha inhibitor 2 antibody (ab20392, 1:500; abcam, UK), the Phospho-AKT (Thr308) Antibody (9275, 1:1,000; Cell Signaling, USA), the Phospho-AKT (Ser473) Antibody (9271, 4060, 1:1,000; Cell Signaling, USA), the AKT antibody (9292, 1:1,000; Cell Signaling, USA), and the PTEN Antibody (9552, 1:1,000; Cell Signaling, USA); The GAPDH (14C10) Rabbit mAb (2118, 1:1,000; Cell Signaling, USA), the β-Actin (13E5) Rabbit mAb (4970, 1:1,000; Cell Signaling, USA), and the β-Tubulin (9F3) Rabbit mAb (2128, 1:1,000; Cell Signaling, USA) were used as internal control proteins. Chemiluminescent signal was detected by ECL staining (Cwbiotech, Beijing, China).

### Transwell migration assay

For the cell transwell migration assay, cells were starved overnight and harvested by trypsinization, then resuspended in 1%FBS media (RPMI-1640 or DMEM) prior to aliquoting 200 μl of cells (1 × 10^5^ cells) onto each upper chamber of the eight μm pore size culture inserts (Corning 3422, NY, USA), which had been pre-placed into the wells of 24-well culture plates. In the lower chambers, 900μl of media supplemented with 10% FBS, was added as a chemoattractant. After incubation for 12-24 h at 37 °C with 5% CO_2_. Cells that had migrated through the pores were fixed with 4% paraformaldehyde for 30 min and stained with 0.1% crystal violet for a further 20 min at room temperature. The chambers were washed twice with PBS both before and after applying the dye. After fixation and staining, Cells that had migrated or invaded through the membrane were imaged through a microscope (Olympus Corp., Tokyo, Japan).

### Cell proliferation assay

For the cell proliferation assay, the Cell Counting Kit-8 (CCK-8; Dojindo Chemical Laboratories, Kumamoto, Japan) was used to determine the number of living cells. We seeded 1 × 10^3^–6 × 10^3^ cells per well after 24 h or 48 h of transfection in 96-well microplates and incubated them for various periods of time (6-8 h, 24 h, 48 h, or 72 h) in normal or special culture conditions. We tested one 96-well plate every 24 h using the CCK-8 assay. The number of viable cells was measured by adding 10 μl of CCK-8 reagent to each well, and incubating at 37 °C for another 2 h, then measuring the absorbance/optical density (OD) at a wavelength of 450 nm using a Bio-Rad imark™ microplate absorbance reader (Bio-Rad Laboratories, Hercules, CA, USA).

### Cell viability

The cell viability of SKOV3 and SKOV3/DDP cells treated with DDP was assessed using the CCK8 assay. The cells were cultured in 96-well microplates at a density of 1 **×** 105 cells per well as described above, but with different concentrations (0, 2, 3, 4, 5, 6, 7, 8, 9 and 10 μM) of cisplatin (Sigma-Aldrich, St. Louis, Missouri, USA). After 24 hours of treatment, CCK-8 (10 μl) was added into each well, and the OD450 was assessed. The cell viability was then calculated as follows:

Cell Viability(%) = [(ODs-ODb)/(ODc-ODb)] **×** 100%. Where ODs, ODb and ODc was the OD value of cell group with DDP, cell group without DDP and blank group(no cells), respectively.

### Cell cycle analysis

For cell-cycle analysis using flow cytometry, FACS analysis was performed as follows: Transfected cells were seeded in 6-well plates, then after 48 h incubation, cells were washed twice with phosphate-buffered saline (PBS), trypsinized, and then centrifuged at 1000 **×** g for 5 min. The harvested cells were re-washed twice with PBS, fixed with 1 ml pre-cooling 70% ethanol, vortexed, and after being incubated at 4°C overnight, stored at −20°C for later use. Prior to testing, cells were taken out of the freezer and put on ice for thawing. After centrifugation, the supernatant was discarded. Approximately 200 μl of Solution B was added and mixed well by hand. Next, cells were incubated at room temperature for 10 min followed by the addition of 200 μl Solution C, then placed both on ice and in the dark for a further 10 min. Cells were analyzed using a FACS Calibur system (BD Biosciences, San Jose, CA, USA).The DNA distribution was measured for 4 h by flow cytometry.

### Construction of luciferase reporter vector

The full-length 3’-UTR of GNAI2, which contains the predicted binding sites (positions 838–844 of GNAI2 3’-UTR:ATGTAGC), was cloned into psiTM-Check2-control vector by GenePharma (Shanghai, China) to generate a wild-type luciferase reporter vector. Then the seed sequence was replaced by TACATCGA for mutagenesis to generate a mutant-type luciferase reporter vector, as its negative control.

### Luciferase reporter assay

For the reporter assays, HEK-293T cells were cultured in 24-well plates and transfected with both the psi™-Check2-GNAI2-3’-UTR WT / psi™-Check2-GNAI2-3’-UTR MUT plasmids (GenePharma, Suzhou, China) at 50 ng and the miR-222-3p mimic/miRNA-control mimic at 50 nM per well, according to the Lipofectamine 2000 transfection system protocol. After 24 h of incubation, cells were lysed using 1 **×** PLB, and transfered into 96-well plates (Nunc™, Thermo Fisher Scientific, Danmark), Firefly and Renilla luciferase activities were measured using the Dual-Luciferase® reporter assay system (Promega, Madison, WI, USA) according to the manufacturer's instructions. Luciferase signal ratio (Rluc/luc) was calculated for each construct.

### Clone of the human GNAI2 cDNA fragment

The GNAI2 primers were designed with reference to the GenBank sequence of guanine nucleotide-binding protein G(i) subunit alpha-2 isoform 2 (accession No. NM_001166425.1). The primers were ligated to the XhoI and EcoRI (New England Biolabs (NEB), Beijing, China) restriction sites using Buffer 2.1 (NEB, Beijing, China). The human GNAI2 cDNA fragment was obtained by qRT-PCR from total RNA of human EOC cells using TRIzol reagent (Invitrogen, CA). The forward primer was 5′-aaCTCGAGatgagaggtgctggg gagtcaggg-3′, and the reverse primer was 5′-ccgGAATTC gaagaggccgcagtccttcaggttg-3′.

### Construction and identification of the pEGFP-N1-GNAI2 expression vector

The pEGFP-N1 vector (Genebank Accession #U55762, given by Professor Qian Feng from the School of Life Sciences, Fudan University, Shanghai, China) and purified GNAI2 cDNA fragment were digested using the XhoI and EcoRI restriction enzymes and after purification, they were ligated by the T4 DNA ligase (Thermo Scientific, MA, USA) to construct the pEGFP-N1-GNAI2 eukaryotic expression vector. The pEGFP-N1-GNAI2 DNA was transformed into Escherichia coli DH5α (CWBIO, Beijing, China) competent cells. White single colonies were collected, and a minor fraction was used for DNA agarose gel electrophoresis. The remaining colonies were placed into kanamycin resistant liquid LB culture medium, and incubated overnight at 37°C on a shaking table. Finally, full length ORF of pEGFP-N1-GNAI2 were sequenced verified by the Beijing Genome Institute.

### Target analysis

Bioinformatic analysis was performed by using these specific programs: Targetscan (release 7.0, http://www.targetscan.org/), Pictar (http://pictar.mdc-berlin.de/), and miRDB (http://www.mirdb.org/miRDB/).

### Statistical analysis

All reactions were performed at least three times and each independent experiment was carried out in duplicate or triplicate for each condition according to the manufacturer's instructions. All statistical analyses were performed using either PASW Statistics 18.0 (SPSS) (Chicago, IL, USA), Excel (Microsoft) and/or GraphPad Prism 5 software (GraphPad Software, Inc., La Jolla, CA, USA). Data from three or more independent experiments are presented as the mean ± the standard deviation. Enumerated data were subjected to a two-sample Student's *t-test* to determine significance, while the measurement data were assessed by an *X*^2^ test.

Relative expression levels of miR-222-3p were characterized by their median ranges. The correlation between the expression of miR-222-3p and clinicopathological characters was assessed with the two-sample Student's *t* test. The Kaplan-Meier method was used for survival analysis and differences in survival were estimated using the Log-Rank test. Univariate Cox regression was performed on each clinical covariate to examine its influence on patient overall survival. Final multivariate models were based on step-wise addition. The association between miR-222-3p and GNAI2 mRNA expression was assessed by means of Pearson's correlation analysis using the Pearson correlation coefficient *r*. All tests were two tailed and data were considered statistically significant if the *P value* was less than 0.05 (*P < 0.05; **P < 0.01; ***P < 0.001).

## SUPPLEMENTARY MATERIALS


