# Supplementary material for: MicroRNA-222-3p/GNAI2/AKT axis inhibits epithelial ovarian cancer cell growth and associates with good overall survival
Source: Oncotarget. 2016 Nov 2;7(49):80633–54. doi: 10.18632/oncotarget.13017 (PMC5348346; doi:10.18632/oncotarget.13017)
Supplement: Supplementary file 1 [file oncotarget-07-80633-s001.pdf]

MicroRNA-222-3p/GNAI2/AKT axis inhibits epithelial ovarian cancer cell growth and associates with good overall survival

Supplementary Materials

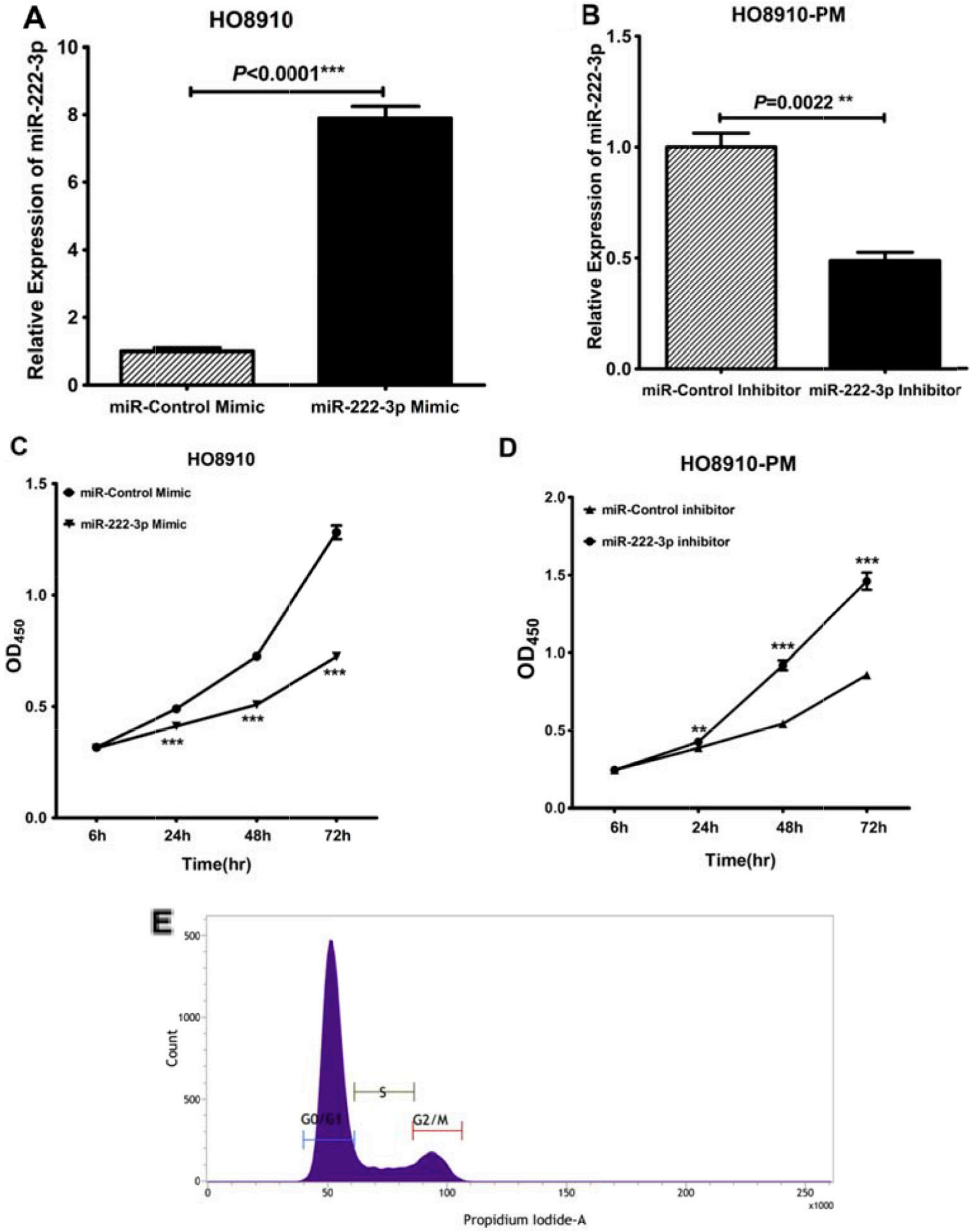

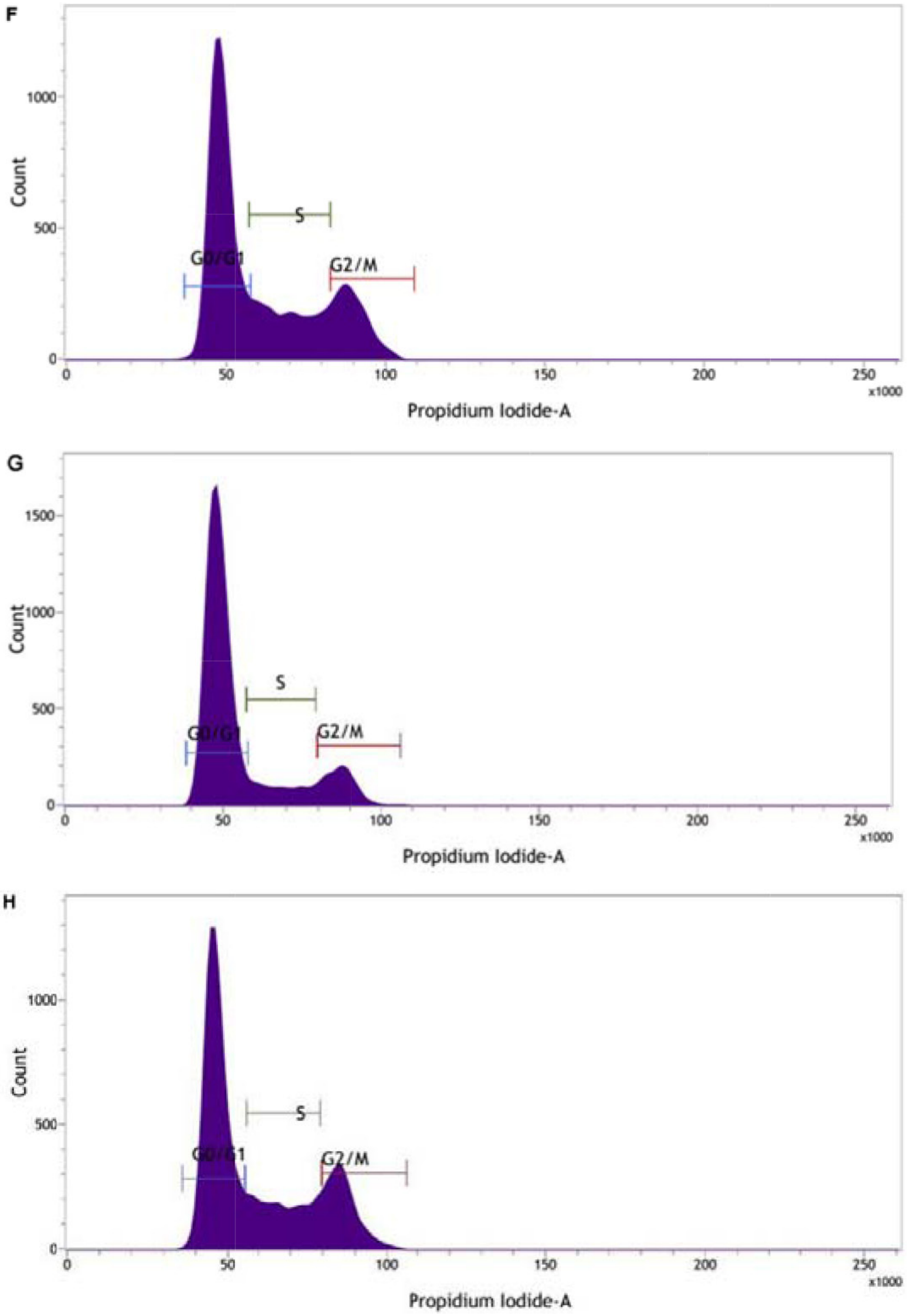

Supplementary Figure S1: MiR-222-3p overexpression decreases EOC cell proliferation and migration in vitro. (**A**) Transfection efficiency of miR-222-3p mimic in HO8910 by qRT-PCR 48h after transfection, using miR-control mimic as a negative control. (**B**) Transfection efficiency of miR-222-3p inhibitor in HO8910-PM by qRT-PCR 48 h after transfection, using miR-control inhibitor as a negative control. (**C**) Cell proliferation was analyzed using a CCK-8 assay. The proliferation of HO8910 transfected with miR-222-3p mimic was reduced, compared with that transfected with miR-control mimic. (**D**) Cell proliferation was analyzed using a CCK-8 assay. The proliferation of HO8910-PM transfected with miR-222-3p inhibitor was reduced, compared with that transfected with miR-control inhibitor. (**E–F**) Cell DNA content distribution of SKOV3/DDP cells transfected with miR-control mimic (E) or miR-222-3p mimic (F) in each phase. (**G–H**) Cell DNA content distribution of SKOV3 cells transfected with miR-control inhibitor (G) or miR-222-3p inhibitor (H) in each phase. Representative images were shown. All values were Mean ± SD, *$P < 0.05$, **$P < 0.01$, ***$P < 0.001$.

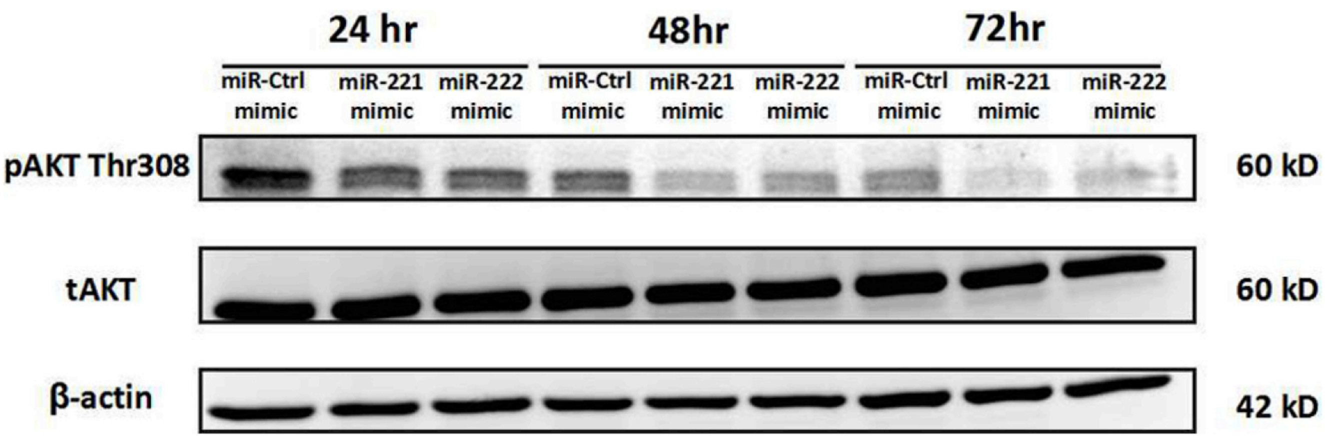

Supplementary Figure S2: MiR-222-3p overexpression reduces ovarian cancer cell proliferation by inhibiting phosphorylation of AKT. Western blot analysis of pAKT (thr308) and total AKT protein levels were detected 24 h, 48 h, and 72 h after transfection of miR-221-3p mimic, miR-222-3p mimic or miR-control mimic in Tara R182 cells.

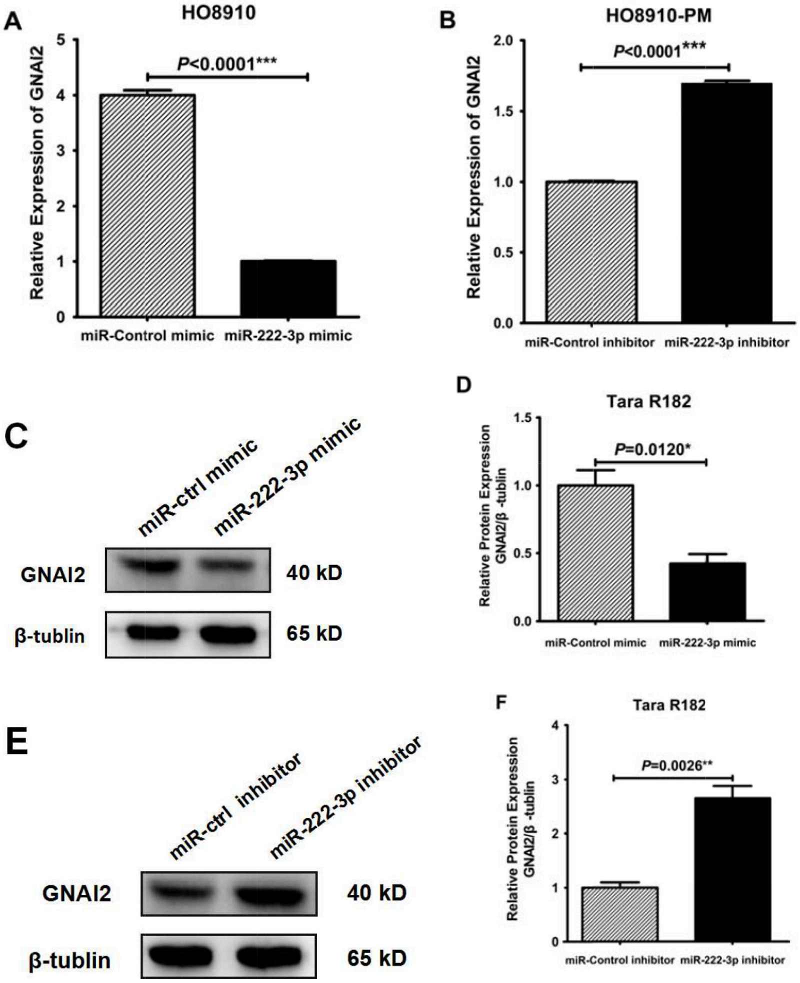

Supplementary Figure S3: MiR-222-3p can decrease EOC cell growth in vitro. (**A**) Relative expression of GNAI2 mRNA in HO8910 transfected with miR-222-3p mimic or miR-control mimic. (**B**) Relative expression of GNAI2 mRNA in HO8910-PM transfected with miR-222-3p inhibitor or miR-control inhibitor. (**C–D**) Western blot assay of the GNAI2 protein levels in Tara R182 transfected with miR-222-3p mimic or miR-control mimic, and the relative expression of GNAI2 was normalized to β-tublin (D). (**E–F**) Western blot assay of the GNAI2 protein levels in Tara R182 transfected with miR-222-3p inhibitor or miR-control inhibitor, and the relative expression of GNAI2 was normalized to β-tublin (F). All values were Mean ± SD, *$P < 0.05$, **$P < 0.01$, ***$P < 0.001$.
